# Exploring the Relationship Between Community-Level Economic Deprivation and HIV Infection Among Hospital Admissions in Washington, DC

**DOI:** 10.7759/cureus.37236

**Published:** 2023-04-07

**Authors:** Oluwasegun A Akinyemi, Ofure V Omokhodion, Mojisola E Fasokun, Oluwafemi E Makanjuola, Sabrina Aaron, Kindha Elleissy Nasef, Martins Chidi, Bukola O Agboola, Tom Ogungbemi, Temitayo Ogundipe, Otolorin Abiodun

**Affiliations:** 1 Health Policy and Management, University of Maryland School of Public Health, College Park, USA; 2 Surgery, Howard University, Washington, DC, USA; 3 Obstetrics and Gynecology, University College Hospital, Ibadan, NGA; 4 Epidemiology and Public Health, University of Alabama at Birmingham, Birmingham, USA; 5 Medicine and Surgery, University of Ilorin, Ilorin, NGA; 6 Obstetrics and Gynecology, Howard University College of Medicine, Washington, DC, USA; 7 Hospital Medicine, Howard University College of Medicine, Washington, DC, USA; 8 Institute of Applied Environmental Health, University of Maryland School of Public Health, College Park, USA; 9 Epidemiology, DC Department of Public Health, Washington, DC, USA; 10 Community and Family Medicine, Howard University Hospital, Washington, DC, USA; 11 Family and Community Medicine, Howard University College of Medicine, Washington, DC, USA

**Keywords:** epidemiology, association, distressed community index, neighborhood socioeconomic distress, hiv incidence

## Abstract

Introduction

Human immunodeficiency virus (HIV) infection is a significant health concern in the United States, affecting 38 million Americans. Despite a recent decline in prevalence, social determinants of health remain an important factor driving infections, particularly among minority populations. However, the relationship between community-level economic deprivation indices and HIV infection among hospital admissions has been understudied in the literature.

Objectives

This study investigated the association between community-level economic deprivation, measured by the Distressed Community Index (DCI), and HIV infection among hospital admissions in Washington, District of Columbia (DC).

Methods

We utilized data from the State Inpatient Database (SID) for Washington, DC, between 2016 and 2019, identifying all admissions with a history of HIV. The multivariate analysis determined the association between DCI quintiles and HIV infection among hospital admissions. Also included in the multivariate analysis were patients' age, sex, race/ethnicity, insurance type, smoking status, obesity, sexually transmitted infections (STIs), hepatitis B infections, and mental health conditions.

Results

Of the 213,682 admissions captured in the DCI quintiles, 67.4% were Black, 17.2% were White, and 10.7% were Hispanic. The prevalence of HIV infection in the study population was 4.4%. There was a statistically significant association between the DCI quintiles and HIV infection among hospital admissions. The residents of the richest neighborhoods defined as prosperous quintile (also the reference group) had the lowest odds of HIV infections compared to the other quintiles (comfortable, odds ratio {OR}=1.94 and 95% confidence interval {CI}=1.38-2.74; mid-tier, OR=1.49 and 95% CI=1.04-2.14; at risk, OR=1.75 and 95% CI=1.22-2.49; and distressed, OR=1.97 and 95% CI=1.38-2.82). Other significant predictors of HIV infection were Black race (OR=1.82; 95% CI=1.41-2.33), age between 45 and 65 years (OR=1.55; 95% CI=1.32-1.80), male sex (OR=1.58; 95% CI=1.40-1.77), and depression (OR=1.21; 95% CI=1.03-1.43).

Conclusion

This study reveals a significant association between increased levels of economic distress and the prevalence of HIV among hospital admissions in Washington, DC. Our findings emphasize the importance of taking social determinants of health into account when addressing HIV prevention and management. Implementing targeted interventions and resources in economically distressed communities may be crucial for reducing HIV prevalence and improving health outcomes for affected populations.

## Introduction

Human immunodeficiency virus (HIV)/AIDS is a significant global health challenge, with approximately 37.7 million people living with HIV at the end of 2020 [1]. In the United States, an estimated 1.2 million people are living with HIV, with approximately 36,801 new diagnoses reported in 2019 [2].

Hospital admissions among HIV-positive individuals have a considerable impact on healthcare systems. A study conducted in the United States between 2000 and 2010 showed that approximately 1.8 million HIV-positive patients were admitted to hospitals, with an admission rate of 25.6 per 100 persons living with HIV [3]. Furthermore, the hospitalization rate for HIV-positive patients was 3.3 times higher than for those without HIV [4]. The primary reasons for hospital admissions among HIV-positive patients included antiretroviral therapy (ART) complications, opportunistic infections, and other comorbidities [5].

Understanding the prevalence of HIV among hospital admissions is essential, as it can inform healthcare providers and policymakers on allocating resources and developing targeted interventions to improve patient outcomes [6]. With the advancement of ART, HIV has become a manageable chronic condition; however, it remains crucial to address the unique healthcare needs of HIV-positive patients to ensure optimal treatment and management [7].

Neighborhood-level economic deprivation plays a significant role in shaping the prevalence of HIV among hospitalized patients in the United States [8]. Although neighborhoods cannot directly cause HIV transmission, they have considerable psychological and social impacts on residents, influencing individual behaviors and either increasing or decreasing vulnerability to HIV [9]. Residents in economically deprived neighborhoods often face limited access to resources, such as healthcare, education, and social support services [10]. These limitations contribute to a higher prevalence of risky behaviors, including unprotected sex, drug use, and engagement in transactional sex, which elevate the risk of HIV transmission [11].

Although current literature has explored the influence of social determinants of health on the prevalence of HIV among hospital patients, a research gap remains in understanding the association between community-level economic deprivation and this particular outcome.

This study aims to examine the relationship between community-level economic deprivation and the prevalence of HIV among hospital patients in Washington, District of Columbia (DC).

## Materials and methods

Data source and patient selection

This study utilized the DC State Inpatient Database (SID) from January 2016 to December 2019 to obtain data on HIV infections among hospitalized patients. The SID is an essential component of the Healthcare Cost and Utilization Project's longitudinal data system, which provides comprehensive hospital care data in the United States. These state-specific resource files include a comprehensive set of inpatient care records that cover over 95% of all hospital discharges in America. These databases are crucial for analyzing national trends in healthcare access, quality, and outcomes [12]. The patient discharge information in the SID was de-identified and did not require institutional review board (IRB) approval.

This study investigated the relationship between community-level economic deprivation using the Distressed Community Index (DCI) and the prevalence of HIV infection among hospital admissions in Washington, DC. The Economic Innovation Group developed the DCI to evaluate the well-being of communities in the United States based on seven metrics. These parameters assess population socioeconomic deprivation based on median income ratio, education level, housing vacancy rates, poverty rate, business establishments, unemployment, and job growth [13]. Education level is determined by the number of residents over 25 years without a high school diploma or an equivalent degree, while the poverty rate is determined by those living below the national poverty threshold. Unemployment refers to community members who are not working (either not in the labor force or retired). The housing vacancy ratio refers to unoccupied units after adjusting for recreational or occasional use. The neighborhood median income is measured against the state median income, job growth is measured against changes in employment, and business establishments are measured against changes in the number of enterprises in the community. These DCI-weighted variables add up to a raw score ranging from 0 (no distress) to 100 (severe distress) based on each area's Zone Improvement Plan (ZIP) code. With further normalization, the scores can be ranked by percentiles that fall into one of the five tiers of socioeconomic distress (prosperous, comfortable, mid-tier, at risk, and distressed).

Dependent variable/study outcome

The primary outcome of this study was the prevalence of HIV among hospital admissions during the study period. The International Classification of Diseases-10 (ICD-10) diagnosis codes were utilized to identify all inpatient admissions with an HIV diagnosis. The dependent variable is binary, with a score of 1 for coexisting HIV infection during hospital admission and 0 for cases without the condition. We controlled for covariates such as patients' age, race/ethnicity, insurance type, and income. Also included in the multivariate analysis are patients' smoking status, alcohol addiction, substance abuse, and mental health conditions such as depression, schizophrenia, and other psychotic disorders and bipolar disorders.

Independent variables

There were 213,682 admissions captured in the DCI quintiles in the DC SID from 2016 to 2019, excluding patients with missing data. Patients' age, sex, race/ethnicity, insurance type, alcohol addiction, substance abuse, and other preexisting comorbidities such as diabetes, obesity, and hypertension were included in the study. Patients' mental condition was also included in the study design, as categorized by ICD-10 (depression, schizophrenia, and other psychotic disorders and bipolar disorders).

Statistical analysis

Statistical analysis involves descriptive statistics techniques such as percentages and frequencies to describe the study variables. We utilized the Pearson chi-square tests to evaluate the association between patients' baseline characteristics and HIV infections among hospital admissions. The multivariate regression analysis obtained adjusted odds ratios (ORs) with 95% confidence intervals (CI). The statistical significance of the results was evaluated using a two-tailed p-value of <0.05. All statistical analyses were conducted using Stata 14 (StataCorp LLC, College Station, TX).

## Results

Table 1 presents the association between the Distressed Community Index and patient characteristics. The residents of prosperous neighborhoods are predominantly young (46.4%) and White (54.5%) and utilize private insurance (43.6%). These patients also exhibit lower rates of smoking (9.7%), HIV prevalence (1.6%), obesity (3.7%), alcohol addiction (3.0%), bipolar disorder (2.9%), and hepatitis (0.2%). On the other hand, residents of communities with the highest neighborhood deprivations, while also young (46.7%), were mainly Black (77.7%), with 43.4% utilizing Medicaid and 23.6% being current smokers. They also have higher HIV prevalence (5.7%), substance abuse (0.04%), and bipolar disorders (5.9%).

**Table 1:** Baseline characteristics stratified across the different quintiles of the Distressed Community Index (DC SID, 2016-2019) P<0.05, statistical significance DC, District of Columbia; SID, State Inpatient Database; HIV, human immunodeficiency virus; STI, sexually transmitted infection

Variables	Total population	Prosperous	Comfortable	Mid-tier	At risk	Distressed	P-value
	N=213,682	n=16,766	n=70,375	n=51,605	n=40,412	n=34,524	
Age							<0.001
18-45 years	46.06%	40.60%	46.44%	47.09%	45.80%	46.70%	
45-65 years	29.25%	17.82%	30.73%	24.59%	33.05%	34.30%	
>65 years	24.69%	41.57%	22.84%	28.32%	21.15%	19.00%	
Female	57.82%	58.05%	56.11%	58.34%	58.48%	59.63%	<0.001
Race/ethnicity							<0.001
White	17.23%	54.53%	26.21%	12.66%	5.57%	1.70%	
Black	67.38%	29.68%	61.82%	67.80%	82.92%	77.65%	
Hispanics	10.65%	7.63%	6.16%	13.24%	8.98%	19.36%	
Others	4.74%	8.17%	5.81%	6.29%	2.52%	1.29%	
Insurance							<0.001
Private	33.59%	43.61%	39.38%	31.34%	27.69%	27.20%	
Medicare	30.25%	40.61%	28.29%	31.67%	29.34%	28.08%	
Medicaid	34.46%	14.31%	30.40%	35.31%	41.18%	43.39%	
Uninsured	1.22%	1.10%	1.34%	1.23%	1.25%	1.02%	
Others	0.48%	0.37%	0.59%	0.44%	0.54%	0.31%	
Current smokers	17.38%	9.72%	17.63%	15.27%	18.11%	23.26%	<0.001
HIV	4.35%	1.56%	4.78%	3.10%	5.19%	5.71%	<0.001
Depression	8.78%	10.97%	9.43%	7.54%	8.90%	8.11%	<0.001
Obesity	5.06%	3.73%	4.62%	5.64%	5.52%	5.16%	<0.001
Substance abuse	0.02%	0.01%	0.02%	0.02%	0.02%	0.04%	0.02
Alcohol addiction	4.95%	2.95%	5.70%	4.02%	5.39%	5.30%	<0.001
Schizophrenia	0.49%	0.23%	0.50%	0.51%	0.55%	0.48%	<0.001
Bipolar	4.74%	2.92%	5.34%	3.42%	5.12%	5.91%	<0.001
Hepatitis B	0.29%	0.20%	0.32%	0.31%	0.27%	0.27%	0.09
STI	0.61%	0.38%	0.58%	0.62%	0.73%	0.65%	<0.001

Table 2 displays the distribution of patient characteristics among those diagnosed with HIV. Although most patients were under 50 (48.8%), most patients with HIV were in the 45-65 age bracket (58.2%). While Blacks constituted 52.6% of the total population, they were overrepresented in the HIV group at 78.1%. Additionally, although 42.9% of the population utilized private insurance, most people with HIV utilized Medicaid insurance (44.8%). Patients with HIV were predominantly smokers (26.3%) and exhibited a higher rate of depression (16.0%), which is almost double the rate of depression in the general population. Patients with HIV were however less likely to be obese (3.9% versus 5.4%) than the general population. Patients with HIV also had higher rates of other mental health conditions such as bipolar disorders (10.1% versus 3.3%), schizophrenia (0.7% versus 0.3%), substance abuse (0.06% versus 0.03%), alcohol addiction (7.7% versus 3.5%), and hepatitis (2.6% versus 0.3%).

**Table 2:** Association between study variables and human immunodeficiency virus (HIV) infection (DC SID, 2016-2019) P<0.05, statistical significance DC, District of Columbia; SID, State Inpatient Database; STI, sexually transmitted infection

Variables	Total	HIV	No HIV	P-value
Age	N=213,682	n=14,772	n=198,910	<0.001
18-45	48.84%	32.66%	49.31%	
45-65	27.00%	58.20%	26.08%	
>65	24.16%	9.14%	24.61%	
Female	55.55%	40.91%	55.98%	<0.001
Race/ethnicity				<0.001
White	28.29%	7.79%	28.89%	
Black	52.62%	78.07%	51.87%	
Hispanics	12.43%	12.54%	12.43%	
Others	6.66%	1.61%	6.81%	
Insurance				<0.001
Private	42.94%	21.90%	43.55%	
Medicare	28.58%	32.30%	28.47%	
Medicaid	26.15%	44.79%	25.61%	
Uninsured	1.26%	0.62%	1.28%	
Others	1.07%	0.38%	1.09%	
Current smokers	14.13%	26.28%	13.63%	<0.001
Depression	8.27%	15.95%	8.04%	<0.001
Obesity	5.36%	3.93%	5.40%	<0.001
Substance abuse	0.02%	0.06%	0.02%	<0.001
Alcohol addiction	3.54%	7.74%	3.41%	<0.001
Schizophrenia	0.33%	0.72%	0.32%	<0.001
Bipolar	3.28%	10.12%	3.08%	<0.001
Hepatitis	0.25%	2.06%	0.20%	<0.001
STI	0.47%	3.03%	0.39%	<0.001

Table 3 highlights the risk factors for HIV in the datasets. Table 3 presents a multivariate regression analysis examining predictors of human immunodeficiency virus (HIV) infection among hospitalized patients. Patients aged 45-65 had higher odds of HIV infection compared to patients aged <45 (reference group). Black and Hispanic patients had higher odds of HIV infection compared to White patients (reference group), with ORs of 1.82 (95% CI=1.41-2.33) and 1.39 (95% CI=1.02-1.91), respectively. Patients with Medicare and Medicaid also had higher odds of HIV infection compared to those with private insurance (reference group). Patients from mid-tier, at-risk, and distressed communities had higher odds of HIV infection compared to those from prosperous communities (reference group), with ORs of 1.49 (95% CI=1.04-2.14; p<0.001), 1.75 (95% CI=1.22-2.49), and 1.97 (95% CI=1.38-2.82), respectively. Also, the odds of HIV infection for patients from comfortable communities were higher than those from prosperous communities (OR=1.94; 95% CI=1.38-2.74). Current smokers had slightly higher odds of HIV infection compared to nonsmokers (OR=1.15; 95% CI=1.00-1.32; p=0.05). Patients with depression had higher odds of HIV infection (OR=1.21; 95% CI=1.03-1.43). Obesity and alcohol addiction were associated with lower odds of HIV infection, with ORs of 0.48 (95% CI=0.36-0.65) and 0.62 (95% CI=0.48-0.82), respectively. The odds of HIV infection were not significantly different for patients with schizophrenia (OR=0.51; 95% CI=0.19-1.39).

**Table 3:** Risk factors for human immunodeficiency virus (HIV) infections (DC SID, 2016-2019) P<0.05, statistical significance DC, District of Columbia; SID, State Inpatient Database; STI, sexually transmitted infection; CI, confidence interval

HIV	Odds ratio	Lower CI	Upper CI	P-value
Age				
18-45	Reference			
45-65	1.55	1.32	1.8	<0.001
>65	0.26	0.21	0.33	<0.001
Female	0.63	0.56	0.71	<0.001
Race/ethnicity				
White	Reference			
Black	1.82	1.41	2.33	<0.001
Hispanics	1.39	1.02	1.91	0.04
Others	0.81	0.45	1.45	0.47
Insurance				
Private	Reference			
Medicare	1.72	1.45	2.02	<0.001
Medicaid	1.69	1.43	1.2	<0.001
Uninsured	0.42	0.11	1.74	0.23
Others	0.63	0.15	2.6	0.52
Distressed Community Index				
Prosperous	Reference			
Comfortable	1.94	1.38	2.74	<0.001
Mid-tier	1.49	1.04	2.14	0.03
At risk	1.75	1.22	2.49	<0.001
Distressed	1.97	1.38	2.82	<0.001
Current smokers	1.15	0.99	1.32	0.05
Depression	1.21	1.03	1.43	0.01
Obesity	0.48	0.36	0.65	<0.001
Alcohol addiction	0.62	0.48	0.82	<0.001
Schizophrenia	0.51	0.19	1.39	0.17
Bipolar	1.22	0.98	1.51	0.07
Hepatitis B	4.66	2.83	7.66	<0.001
STI	5.49	3.59	8.39	<0.001

**Figure 1:**
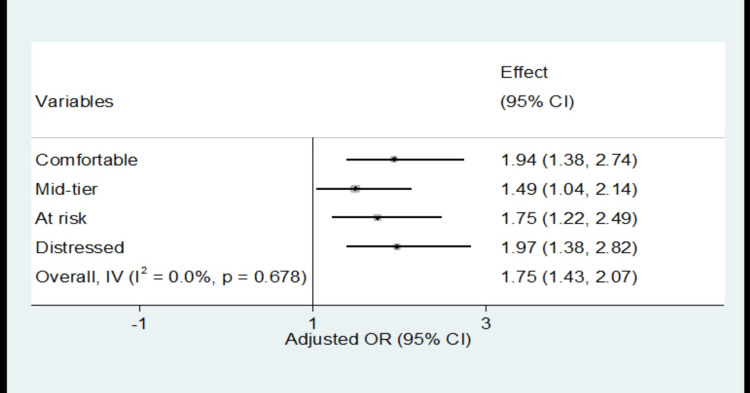
Forest plot showing an independent association between DCI and HIV infection among hospital admissions (DC SID, 2016-2019) The vertical line represents the reference group (residents of prosperous neighborhoods). "Overall, IV" shows the combined effect of all groups, calculated using the inverse variance method. The I^2^ statistic measures the proportion of variability between different DCI quintiles. I^2^=0 and a p-value of 0.678 suggest no statistically significant heterogeneity among the quintiles DC, District of Columbia; SID, State Inpatient Database; HIV, human immunodeficiency virus; CI, confidence interval; OR, odds ratio; DCI, Distressed Community Index; IV, inverse variance

Figure 1 presents a forest plot illustrating the independent association between the Distressed Community Index (DCI) and HIV-related admissions. The plot reveals a positive correlation between increasing levels of socioeconomic deprivation and the rate of HIV-related admissions. This association is particularly pronounced among mid-tier, at-risk, and distressed communities, indicating a stronger relationship between deprivation and HIV-related admissions.

## Discussion

The Distressed Community Index (DCI) is a comprehensive ranking system that utilizes Zone Improvement Plan (ZIP) codes to assess a community's level of socioeconomic distress through seven parameters, which are unemployment, poverty rate, median income, education level, business establishments, housing vacancies, and job growth. As a social determinant of health, the DCI can be utilized to identify communities at risk of health disparities in the United States [13]. Among the 213,652 individuals captured in the Washington, DC, DCI, a significant proportion was Black (67.4%) and female (57.8%), aged 18-45 years (46.1%). Despite a decline in the prevalence of HIV, recent statistics demonstrate that young male Blacks and Hispanics are still significantly affected [14].

Our study found that over half (54.6%) of the prosperous communities were Whites compared to the proportion of Whites in the general DC SID population (17.0%). In contrast, in the distressed communities, the proportion of Whites was only 1.7%, compared to 77.7% of Blacks and 19.4% of Hispanics. This is consistent with prior research, which found that White families had eight times more wealth than Black families and five times that of Hispanic families [15]. Our findings suggest that although most of the population was Black, more Whites lived in prosperous communities, and more Blacks resided in distressed communities, thus confirming socioeconomic segregation.

Among hospitalized patients, the prevalence of HIV was among residents of prosperous communities (1.6%) and highest among residents of at-risk (5.2%) and distressed communities (5.7%). Individuals with HIV were predominantly Black, male, and mainly utilizing public insurance (Medicare or Medicaid) and had higher rates of bipolar disorder, STIs, and hepatitis B infections. This is consistent with prior research showing higher rates of HIV infection among Black and Hispanic males who have sex with males living in poor areas and who lack insurance coverage [16,17]. Poverty may drive the HIV pandemic as individuals in distressed communities may have less access to healthcare or poorer health-seeking behavior and may be more likely to engage in risky sexual behavior [16-18].

After controlling for relevant variables such as age, sex, race/ethnicity, insurance status, and behavioral lifestyle, our study revealed a significant association between geographic economic deprivation, as measured by the Distressed Community Index (DCI), and higher prevalence of HIV among hospitalized patients in Washington, DC. Specifically, we observed that mid-tier, at-risk, and distressed communities had a higher prevalence of HIV among hospital admissions reported in the Washington, DC, State Inpatient Database compared to residents of prosperous neighborhoods. These findings align with previous studies that have reported an increased burden of HIV infection in socioeconomically disadvantaged neighborhoods, where factors such as limited access to healthcare, inadequate housing, and increased levels of substance abuse may contribute to higher HIV transmission rates [16,19].

Interestingly, our study also revealed a similarly high rate of HIV among hospitalized patients residing in comfortable communities. This unexpected finding suggests that there may be additional factors at play that may be unmeasured in the present study. As a result, this particular finding warrants further exploration in subsequent studies to better understand the complex interplay of factors contributing to HIV prevalence in both economically deprived and more affluent communities.

Finally, our findings highlight the varying influence of different risk factors on HIV infection among hospitalized patients. We observed that current smokers and patients with depression had increased odds of HIV infection, with ORs of 1.15 (95% CI=1.00-1.32; p=0.05) and 1.23 (95% CI=1.05-1.45; p=0.01), respectively. In contrast, obesity and alcohol addiction were associated with lower odds of HIV infection, with ORs of 0.48 (95% CI=0.36-0.65; p<0.001) and 0.63 (95% CI=0.48-0.82; p<0.001), respectively. No significant difference was observed in the odds of HIV infection for patients with schizophrenia. These findings underscore the complex interplay of individual risk factors in determining HIV infection risk among hospitalized patients and highlight the need for tailored prevention strategies.

Public health significance

The public health significance of this study is that it highlights the importance of addressing social and structural barriers to care if the spread of HIV is to be curbed. The study also highlights the relationship between neighborhood economic deprivation and HIV prevalence among hospital admissions in Washington, DC. It further emphasizes the importance of including social determinants of health in strategies designed for preventing and managing HIV, particularly among minority populations. Our findings underscore the need for interventions to address structural and social barriers to care in distressed communities, where much of the burden of HIV in Washington, DC, appears to lie. Finally, the study adds to the body of knowledge on HIV and social determinants of health. It can inform policy and public health strategies to reduce the incidence of HIV in the United States.

Limitations and strengths

The study's strengths include a large sample size and robust variables available for analysis, addressing a gap in knowledge regarding the association between DCI and the prevalence of HIV among hospital patients in Washington, DC. However, a limitation is the lack of exploration of sexual orientation or gender identity, which are critical in HIV transmission and spread. Future research should address this gap and explore the association between HIV prevalence and sexual orientation or gender identity. In addition to DCI, other social determinants of health should be considered in developing comprehensive prevention and management strategies for HIV, including access to healthcare, education, income, and housing. These interventions should aim to reduce disparities in access to care and address social and structural barriers that prevent individuals from accessing necessary care. Finally, in the present study, we employed hospital data as the primary source of information. Consequently, the conclusions drawn and their generalizability to the broader community should be interpreted with caution, as the findings may not entirely reflect the overall population's characteristics and experiences. Overall, this study emphasizes the need for a holistic approach to addressing HIV, which includes addressing social determinants of health, targeting interventions toward at-risk communities, and addressing the unique challenges different demographic groups face. These efforts can contribute to reducing the prevalence of HIV and improve health outcomes for all individuals affected by the disease.

## Conclusions

In conclusion, this study provides evidence of a significant association between community-level economic deprivation, measured by the Distressed Community Index (DCI), and the prevalence of HIV among hospital admissions in Washington, DC. The study identified that residents of regions classified as comfortable, mid-tier, at risk, and distressed had a higher prevalence of HIV among hospitalized patients than those in prosperous neighborhoods. Our findings highlight the importance of including social health determinants in strategies designed to prevent and manage HIV. These results underscore the need for tailored interventions to address the socioeconomic factors contributing to the disease's spread.
